# Partial Inhibition of the 6-Phosphofructo-2-Kinase/Fructose-2,6-Bisphosphatase-3 (PFKFB3) Enzyme in Myeloid Cells Does Not Affect Atherosclerosis

**DOI:** 10.3389/fcell.2021.695684

**Published:** 2021-08-12

**Authors:** Renée J. H. A. Tillie, Jenny De Bruijn, Javier Perales-Patón, Lieve Temmerman, Yanal Ghosheh, Kim Van Kuijk, Marion J. Gijbels, Peter Carmeliet, Klaus Ley, Julio Saez-Rodriguez, Judith C. Sluimer

**Affiliations:** ^1^Department of Pathology, Cardiovascular Research Institute Maastricht (CARIM), Maastricht University Medical Center, Maastricht, Netherlands; ^2^Faculty of Medicine, Institute for Computational Biomedicine, Heidelberg University Hospital, Heidelberg University, Heidelberg, Germany; ^3^Institute of Experimental Medicine and Systems Biology, Rheinisch-Westfälische Technische Hochschule (RWTH) Aachen University, Aachen, Germany; ^4^La Jolla Institute for Immunology, San Diego, CA, United States; ^5^Department of Pathology, GROW-School for Oncology and Developmental Biology, Maastricht University Medical Center, Maastricht, Netherlands; ^6^Department of Medical Biochemistry, Experimental Vascular Biology, Amsterdam UMC, University of Amsterdam, Amsterdam, Netherlands; ^7^Laboratory of Angiogenesis and Vascular Metabolism, Department of Oncology, Center for Cancer Biology, Vlaams Instituut voor Biotechnologie (VIB), Leuven Cancer Institute, KU Leuven, Leuven, Belgium; ^8^State Key Laboratory of Ophthalmology, Zhongshan Opthalmic Center, Sun Yat-sen University, Guangzhou, China; ^9^Department of Biomedicine, Aarhus University, Aarhus, Denmark; ^10^Department of Bioengineering, University of California, San Diego, San Diego, CA, United States; ^11^British Heart Foundation (BHF) Centre for Cardiovascular Sciences (CVS), University of Edinburgh, Edinburgh, United Kingdom

**Keywords:** myeloid cells, PFKFB3, macrophage, dendritic cell, glycolysis, atherosclerosis, neutrophil, glycolysis inhibition

## Abstract

**Background:**

The protein 6-phosphofructo-2-kinase/fructose-2,6-bisphosphatase-3 (PFKFB3) is a key stimulator of glycolytic flux. Systemic, partial PFKFB3 inhibition previously decreased total plaque burden and increased plaque stability. However, it is unclear which cell type conferred these positive effects. Myeloid cells play an important role in atherogenesis, and mainly rely on glycolysis for energy supply. Thus, we studied whether myeloid inhibition of PFKFB3-mediated glycolysis in *Ldlr^–/–^LysMCre^+/–^Pfkfb3^*fl/fl*^* (*Pfkfb3^*fl/fl*^*) mice confers beneficial effects on plaque stability and alleviates cardiovascular disease burden compared to *Ldlr^–/–^LysMCre^+/–^Pfkfb3^*wt/wt*^* control mice (*Pfkfb3^*wt/wt*^*).

**Methods and Results:**

Analysis of atherosclerotic human and murine single-cell populations confirmed *PFKFB3/Pfkfb3* expression in myeloid cells, but also in lymphocytes, endothelial cells, fibroblasts and smooth muscle cells. *Pfkfb3^*wt/wt*^* and *Pfkfb3^*fl/fl*^* mice were fed a 0.25% cholesterol diet for 12 weeks. *Pfkfb3^*fl/fl*^* bone marrow-derived macrophages (BMDMs) showed 50% knockdown of *Pfkfb3* mRNA. As expected based on partial glycolysis inhibition, extracellular acidification rate as a measure of glycolysis was partially reduced in *Pfkfb3^*fl/fl*^* compared to *Pfkfb3^*wt/wt*^* BMDMs. Unexpectedly, plaque and necrotic core size, as well as macrophage (MAC3), neutrophil (Ly6G) and collagen (Sirius Red) content were unchanged in advanced *Pfkfb3^*fl/fl*^* lesions. Similarly, early lesion plaque and necrotic core size and total plaque burden were unaffected.

**Conclusion:**

Partial myeloid knockdown of PFKFB3 did not affect atherosclerosis development in advanced or early lesions. Previously reported positive effects of systemic, partial PFKFB3 inhibition on lesion stabilization, do not seem conferred by monocytes, macrophages or neutrophils. Instead, other *Pfkfb3*-expressing cells in atherosclerosis might be responsible, such as DCs, smooth muscle cells or fibroblasts.

## Introduction

Myeloid cells [i.e., monocytes, macrophages, neutrophils and dendritic cells (DCs)] play an active role in atherogenesis. Early pathogenesis of atherosclerotic plaques is characterized by activation of intimal endothelial cells (ECs) in arteries, followed by extravasation of low-density lipoprotein (LDL) cholesterol (Tabas et al., 2007). In the subendothelial space, LDL is oxidized (oxLDL) by reactive oxygen species (ROS) and enzymes (Tabas et al., 2007). This results in a pro-inflammatory response that triggers myeloid cell recruitment (Moore and Tabas, 2011; Silvestre-Roig et al., 2020). Recruited myeloid cells act in parallel to stimulate inflammation through cytokine secretion and other mechanisms. Recruited, activated neutrophils further stimulate monocyte recruitment and macrophage activation. Furthermore, neutrophils contribute to the pro-inflammatory environment by secretion of ROS and neutrophil extracellular traps (NETs), and to LDL oxidation by secreting myeloperoxidase (Silvestre-Roig et al., 2020). DCs modulate T cell responses in atherosclerosis. Additionally, recruited monocytes can differentiate into macrophages or monocyte-derived DCs (moDCs), which ingest oxLDL and become lipid-laden foam cells (Moore and Tabas, 2011; Subramanian and Tabas, 2014; Zernecke, 2015). Excess uptake of oxLDL can result in leukocyte apoptosis. In advanced disease stages, accumulation of apoptotic leukocytes in combination with decreased phagocytic clearance contributes to formation of a detrimental necrotic core (Moore and Tabas, 2011). Moreover, during atherogenesis, smooth muscle cells (SMCs) migrate into the plaque and synthesize collagen, forming a stabilizing fibrous cap. Secretion of matrix metalloproteinases, serine proteases and NETs by macrophages and neutrophils can cause fibrous cap thinning (Moore and Tabas, 2011; Silvestre-Roig et al., 2020). This increases the risk of plaque rupture, which can have detrimental consequences.

Activated neutrophils, DCs and pro-inflammatory macrophages highly depend on glycolysis for their energy production and function (Galván-Peña and O’Neill, 2014; Kumar and Dikshit, 2019; Wculek et al., 2019). During glycolysis, glucose is metabolized to pyruvate, yielding ATP and NADH (Lunt and Heiden, 2011). A rate-limiting step of glycolysis is the conversion of fructose-6-phosphate into fructose-1,6-bisphosphate, catalyzed by phosphofructokinase-1 (PFK-1). Another enzyme, 6-phosphofructo-2-kinase/fructose-2,6-bisphosphatase-3 (PFKFB3), catalyzes the conversion of fructose-6-phosphate into fructose-2,6-bisphosphate, which is an allosteric activator of PFK-1. Thus, PFKFB3 is a potent stimulator of glycolytic rate (Lunt and Heiden, 2011), and possibly an attractive target to interfere with myeloid cell function in atherogenesis.

A few studies have indeed assessed the effect of systemic administration of 3-(3-pyridinyl)-1-(4-pyridinyl)-2-propen-1-one (3PO) or derivatives to partially inhibit PFKFB3 in atherosclerosis. These studies reported decreased total plaque burden (Perrotta et al., 2020) and increased plaque stabilization, respectively (Beldman et al., 2019; Poels et al., 2020). However, as these studies entailed systemic pharmacological PFKFB3 inhibition, it is unclear which cell type confers these positive effects. Although *Pfkfb3* expression in atherosclerotic DCs and neutrophils remains to be assessed, Tawakol et al. (2015) reported increased *Pfkfb3* expression in macrophages incubated with atherosclerosis-relevant stimuli *in vitro*. This effect was exacerbated by hypoxia. Still, the *in vivo* effect of partial inhibition of PFKFB3-mediated glycolysis, specifically in myeloid cells, on atherogenesis has not been studied. Thus, we studied the hypothesis that myeloid inhibition of PFKFB3-mediated glycolysis in *Ldlr^–/–^LysMCre^+/–^Pfkfb3^*fl/fl*^ (Pfkfb3^*fl/fl*^)* mice confers beneficial effects on plaque stability and alleviates cardiovascular disease burden compared to *Ldlr^–/–^LysMCre^+/–^Pfkfb3^*wt/wt*^* control mice (*Pfkfb3^*wt/wt*^).*

## Materials and Methods

### Single-Cell Gene Expression Analysis

Single-cell RNA-sequencing (scRNA-seq) datasets from atherosclerotic plaques were collected from Gene Expression Omnibus (GEO) database or requested to corresponding authors: Wirka et al., 2019 (4 human specimens, GSE131780), Zernecke et al., 2020 (meta-analysis from 9 mice datasets), and van Kuijk et al., 2021 (11 pooled *Ldlr^–/–^ LysMCre^+/–^* mice, GSE150089). Seurat R package (v3.0.1) was used as toolbox for analysis (Stuart et al., 2019) in R (v3.6.1). Single-cell gene expression was normalized by library size, multiplied by a scaling factor of 10,000 and log-transformed. Original cell cluster annotations were used for analysis. *HIF1*α*/Hif1*α (hypoxia-inducible factor 1-alpha) transcription factor (TF) activity was estimated using DoRothEA^1^ (Garcia-Alonso et al., 2019), using the TF regulons of A, B, and C confidence classes as previously described (Holland et al., 2020). For 2-group comparison between cells undergoing and not undergoing hypoxia response, cells were stratified by the third quartile (Q3) of *HIF1A/Hif1a* TF activity within each cell cluster (high > Q3, low ≤ Q3). Differential *PFKFB3/Pfkfb3* expression was performed using Wilcoxon Rank Sum test. No test was performed when the sample size of any condition was lower than 5 observations. *P*-values were adjusted for multiple testing using the Benjamini and Hochberg method. R effect sizes from Wilcoxon Rank-Sum test were calculated as Z divided by the square root of total observations. The greater the absolute r value, the greater the effect size, with positive values for an effect in cells with High *HIF1A/Hif1a* activity. Dot plots show the percentage of cells within cell clusters that express the gene (size), and average expression of each cluster scaled across clusters. Violin plots show the normalized gene expression level of each cell cluster with individual observations (each cell) as data points, 50th percentile of the distribution as a horizontal line, and sample sizes (number of cells) at the bottom. For 2-group comparisons, violin plots are split by hypoxia response stratification, with Wilcoxon test statistics of FDR-adjusted *p*-values and r effect sizes at the top. Analysis code is available at https://github.com/saezlab/Myeloid_PFKFB3_atherosclerosis.

### Experimental Animals

Mouse experiments were approved by regulatory authorities of Maastricht University Medical Centre and performed in compliance with Dutch governmental guidelines and European Parliament Directive 2010/63/EU on protection of animals used for scientific purposes. Mice with a loxP-flanked *Pfkfb3* gene (*Pfkfb3^*lox/lox*^*) (De Bock et al., 2013) were crossed to mice with both a LDL receptor knockout (*Ldlr^–/–^*) to ensure atherosclerosis susceptibility, and hemizygous Cre-recombinase expression under control of the *Lyz2* gene promoter (*LysMCre*^+/–^). *Lyz2* is highly expressed in macrophages, monocytes and neutrophils, and to a lower extent in DCs (Supplementary Figure 1A; Faust et al., 2000). Thus, myeloid-specific Cre-mediated excision of the *Pfkfb3* gene could be ensured. Resulting mice (*Ldlr^–/–^LysMCre^+/–^Pfkfb3^*fl/fl*^*) are referred to as *Pfkfb3^*fl/fl*^*. *Ldlr^–/–^LysMCre^+/–^Pfkfb3^*wt/wt*^* mice were used as controls (*Pfkfb3^*wt/wt*^*). Mice were housed in the Maastricht University laboratory animal facility under standard conditions, in individually ventilated cages (GM500, Techniplast) with up to 5 animals per cage, with bedding (corncob, Technilab-BMI) and cage enrichment. Cages were changed weekly, reducing handling of mice during non-intervention periods.

### Induction of Atherosclerosis and Tissue Collection

To induce atherosclerosis, 11-week-old male *Pfkfb3^*wt/wt*^* and *Pfkfb3^*fl/fl*^* mice were fed a high cholesterol diet (HCD) for 12 weeks *ad libitum*, containing 0.25% cholesterol (824171, Special Diet Services). Mice were euthanized by intraperitoneal pentobarbital injection (100 mg/kg). Blood was withdrawn from the right ventricle and centrifuged (2,100 rpm, 10 min, 4°C). Plasma aliquots were stored at −80°C. Brachiocephalic arteries (BCAs) and hearts were dissected, fixed in 1% PFA overnight and paraffin-embedded.

### Plasma Cholesterol and Triglyceride Levels

Plasma cholesterol (Cholesterol FS Ecoline, 113009990314; DiaSys Diagnostic Systems GmbH) and triglyceride (FS5’ Ecoline, 157609990314; DiaSys Diagnostic Systems GmbH) levels were assessed by standard enzymatic techniques, automated on the Cobas Fara centrifugal analyzer (Roche).

### Histology and Immunohistochemistry

Paraffin-embedded BCA and aortic root (AR) were serially sectioned (4 μm) and stained with hematoxylin and eosin (H&E) to quantify plaque size and necrotic core content. For ARs, five consecutive H&E sections with 20 μm intervals were blinded and analyzed using computerized morphometry (Leica QWin V3, Cambridge, United Kingdom). The sum of plaque within three valves was averaged per mouse. Total plaque burden was quantified in BCA (Σ total plaque length/Σ total vessel length). Furthermore, AR atherosclerotic plaques were analyzed for macrophage content (MAC3+ area/plaque area, 553322, BD), collagen content (Sirius Red+ area/plaque area, 09400, Polyscience) and neutrophil content (Ly6G+ cells/plaque area, 551459, BD). Antigen retrieval was performed with pepsin digestion (Ly6G) or at pH 6 (MAC3, Target Retrieval Solution, S2031, DAKO). Stainings were analyzed using Leica Qwin software (V3, Cambridge United Kingdom) or QuPath V0.2.3 (Bankhead et al., 2017).

### Isolation and Differentiation of Bone Marrow Cells

Femur and tibia of *Pfkfb3^*wt/wt*^* and *Pfkfb3^*fl/fl*^* mice on standard laboratory diet were dissected. Bones were flushed with PBS and cells passed through a 70 μm cell strainer.

To obtain bone marrow-derived macrophages, bone marrow cells were cultured in RPMI 1640 medium (72400047, Gibco), with 15% L929-conditioned medium, 10% fetal calf serum (FCS, FBS-12A, Capricorn Scientific) and 1% penicillin-streptomycin (15070-063, Gibco). After 7-day differentiation, BMDMs were detached with lidocaine and plated for downstream assays. For pro-inflammatory polarization of BMDMs, cells were incubated with LPS (10 ng/ml, L2880, Sigma) and IFN-γ (100 units/ml, HC1020, Hycult Biotech) for 24 h after overnight attachment.

To obtain bone marrow-derived DCs, bone marrow cells were cultured in IMDM medium (21980032, Thermo Fisher Scientific), with 5% FCS, 0.029 mM 2-mercaptoethanol, 150 ng/ml Flt3 ligand (472-FL, R&D Systems) and 1% penicillin-streptomycin for 8 days. After differentiation, DCs were detached by rinsing.

### Quantitative PCR

RNA was isolated with TRIzol reagent (15596026, Thermo Fisher Scientific) according to manufacturer’s protocol. RNA concentrations were determined by NanoDrop 2000 (Thermo Fisher Scientific) and reverse transcription performed following manufacturer’s protocol (1708890, Bio-Rad and 04379012001, Roche). Real-time qPCR was performed using 10 ng cDNA, SYBR Green Supermix (1708885, Bio-Rad) and specific primer sets (Supplementary Table 1). One housekeeping gene (18s rRNA) was used to correct for different mRNA quantities between samples.

### Lactate and Glucose Levels

Lactate and glucose levels in *Pfkfb3^*wt/wt*^* and *Pfkfb3^*fl/fl*^* BMDM cell culture medium were assessed after 26 h of conditioning, using a GEM Premier 4000 Analyzer and the manufacturer’s protocol (Instrumentation Laboratory).

### Seahorse

BMDMs were plated onto XF96 tissue culture microplates. Growth medium was replaced with glucose-free assay medium (RPMI-1640 (R1383, Sigma), 143 mM NaCl, 3 mg/L Phenol Red, 2 mM L-glutamine, in dH_2_O, pH 7.35) and cells were incubated in a non-CO_2_ incubator for 1 h. Thereafter, the assay was performed according to manufacturer’s protocol (103020-100, Agilent), using a 10 mM glucose stimulus, with a Seahorse XF96 Analyzer (Agilent).

### Statistical Analyses

Data are represented as mean ± SEM. For results besides single-cell analysis, ROUT outlier analysis was performed and subsequently, normality (Shapiro-Wilk) and equal variances (*F*-test) analysis and corresponding parametric or non-parametric testing were performed for two-independent groups. ^∗^*p* < 0.05, ^∗∗^*p* < 0.01, and ^∗∗∗^*p* < 0.001.

## Results

### Expression of *PFKFB3/Pfkfb3* in Human and Murine Plaques in Both Immune and Stromal Cells

**FIGURE 1:**
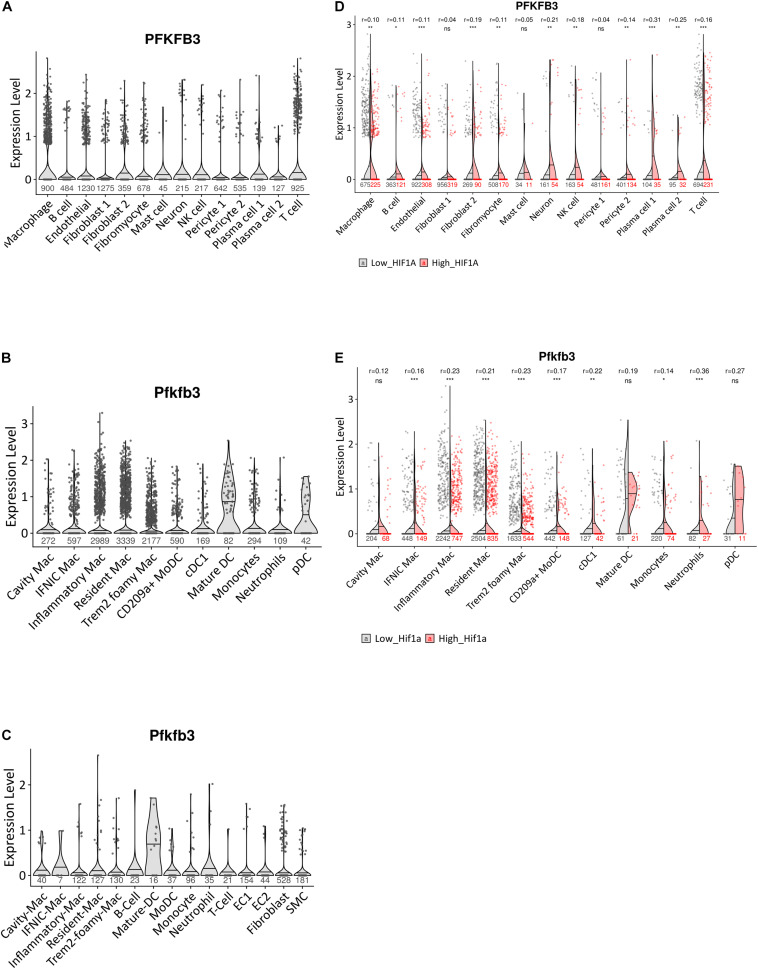
Expression pattern of *PFKFB3/Pfkfb3* in human and murine atherosclerosis. **(A)** Violin plot of *PFKFB3* expression in single-cell populations of human atherosclerotic coronary arteries (Wirka et al., 2019). **(B)** Violin plot of *Pfkfb3* expression in single-cell populations of murine atherosclerotic myeloid cells (Zernecke et al., 2020). **(C)** Violin plot of *Pfkfb3* expression in single-cell populations of murine *Ldlr^– /–^ LysMCre^+/–^* aortic arch lesions (van Kuijk et al., 2021). **(D)** Split violin plot of *PFKFB3* expression in cells with high versus low *HIF1*α signature from human atherosclerotic coronary arteries (Wirka et al., 2019). **(E)** Split violin plot of *Pfkfb3* expression in murine atherosclerotic myeloid cells, with high versus low *Hif1*α signature (Zernecke et al., 2020). In **(D,E)**, Wilcoxon test statistics of FDR-adjusted *p*-values and r effect sizes are indicated at the top. Sample sizes per cell type indicated under (split) violin plots. CD, cluster of differentiation; cDC, conventional dendritic cell; EC, endothelial cell; HIF1α, hypoxia-inducible factor 1-alpha; IFNIC, interferon-inducible; Mac, macrophage; moDC, monocyte-derived dendritic cell; NK cell, natural killer cell; pDC, plasmacytoid DC; SMC, smooth muscle cell; TREM2, triggering receptor expressed on myeloid cells 2. ^∗^*p* < 0.05, ^∗∗^*p* < 0.01, ^∗∗∗^*p* < 0.001.

We first sought to assess *PFKFB3/Pfkfb3* expression patterns in human and murine atherosclerotic plaques. The scRNA-seq dataset from human atherosclerotic coronary arteries by Wirka et al. (2019) showed *PFKFB3* expression mainly in macrophages, but also in ECs, fibroblasts and other leukocytes such as T cells (Figure 1A). This confirms PFKFB3 expression in human atherosclerosis, and particularly in macrophages.

Next, we analyzed murine *Pfkfb3* expression in myeloid cells specifically, from the scRNA-seq meta-analysis by Zernecke et al. (2020) including data from 9 atherosclerosis studies of murine aorta. Traditionally, macrophages were classified into pro-inflammatory M1 and anti-inflammatory M2 macrophages (Mantovani et al., 2009). However, the rise of single-cell techniques has shown that macrophage phenotypes are diverse and has led to identification of 5 main macrophage subsets in atherosclerosis: resident-like macrophages, inflammatory macrophages, foamy “triggering receptor expressed on myeloid cells” (TREM2^*hi*^) macrophages, interferon (IFN)-inducible macrophages, and so-called cavity macrophages, whose transcriptome resembles that of peritoneal macrophages (Willemsen and de Winther, 2020; Zernecke et al., 2020). Furthermore, DCs can be broadly classified into moDCs, plasmacytoid DCs (pDCs) and conventional DCs (cDCs) (Eisenbarth, 2019). Interestingly, of all myeloid cells, *Pfkfb3* expression was highest in mature DCs and pDCs. Furthermore, *Pfkfb3* was expressed across the 5 main macrophage subsets, albeit by a low percentage of cells (Figure 1B). Aside from the aforementioned DC and macrophage subsets, *Pfkfb3* expression was confirmed in monocytes, neutrophils, cluster of differentiation (CD) 209a + moDCs and cDCs.

The meta-analysis dataset contains only myeloid data from several murine atherosclerosis models. Considering the use of *Ldlr^–/–^LysMCre^+/–^* mice in the current study, we confirmed *Pfkfb3* expression in a scRNA-seq dataset from *Ldlr^–/–^LysMCre^+/–^* aortic arch lesions by van Kuijk et al. (2021). Although the number of cells in this *Ldlr^–/–^LysMCre^+/–^* dataset is low, in accordance with the meta-analysis and human datasets, *Pfkfb3* was expressed in a wide range of plaque cells, including macrophage subsets, monocytes, neutrophils, DCs and lymphocytes, but also ECs, SMCs and fibroblasts (Figure 1C).

In line with *in vitro* analysis of HIF1α-dependent expression of *PFKFB3* (Tawakol et al., 2015), *in vivo PFKFB3/Pfkfb3* expression was significantly increased in both human and murine atherosclerotic macrophages with high *HIF1*α*/Hif1*α signatures (Figures 1D,E). However, next to macrophages, a high *HIF1*α*/Hif1*α signature was associated with increased *PFKFB3/Pfkfb3* expression in human ECs, fibroblasts, B cells, neurons, NK cells, pericytes and plasma cells, and in murine CD209a+ moDCs and cDCs. Although cell number is low in some populations, these data suggest that hypoxia regulates *PFKFB3/Pfkfb3* expression *in vivo*, both in humans and mice, in a wide range of cell types.

### Decreased Glycolysis and Pro-inflammatory Profile in *Pfkfb3^*fl/fl*^* Macrophages

**FIGURE 2:**
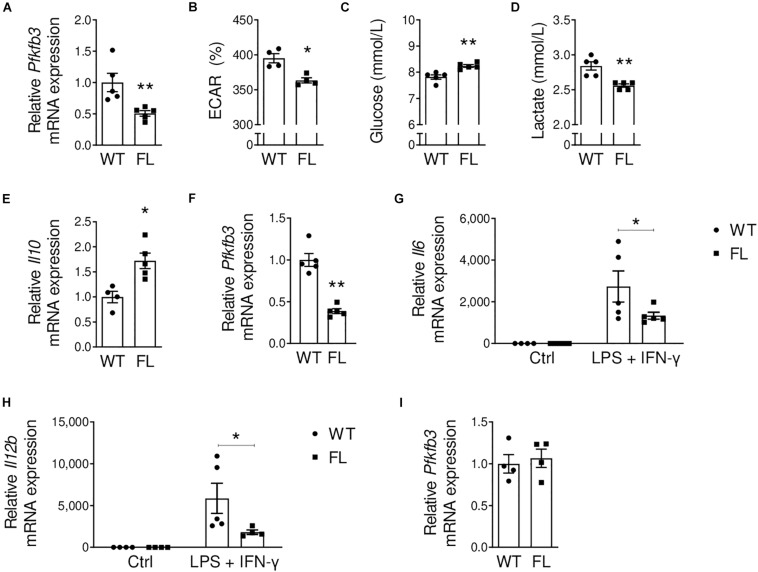
Decreased *Pfkfb3* expression and decreased glycolysis in *Pfkfb3^*fl/fl*^* bone marrow-derived macrophages. **(A)** *Pfkfb3* mRNA expression in *Pfkfb3^*fl/fl*^* (FL) relative to *Pfkfb3^*wt/wt*^* (WT) bone marrow-derived macrophages (BMDM). **(B)** Extracellular acidification rate (ECAR, percentage of baseline) of *Pfkfb3^*fl/fl*^* and *Pfkfb3^*wt/wt*^* BMDMs after 10 mM glucose stimulus, as assessed with a Seahorse XF Analyzer. **(C)** Glucose and **(D)** lactate levels in *Pfkfb3^*fl/fl*^* and *Pfkfb3^*wt/wt*^* BMDM culture media after 26 h of culture. **(E)** *Il10* mRNA expression in unstimulated *Pfkfb3^*fl/fl*^* relative to *Pfkfb3^*wt/wt*^* BMDMs. **(F)** *Pfkfb3* mRNA expression in *Pfkfb3^*fl/fl*^* relative to *Pfkfb3^*wt/wt*^* BMDMs after pro-inflammatory stimulation with LPS and IFN-γ. **(G)** *Il6* and **(H)** *Il12b* mRNA expression in *Pfkfb3^*fl/fl*^* and *Pfkfb3^*wt/wt*^* BMDMs cultured without additions (ctrl; control) or stimulated with LPS and IFN-γ. Expression is relative to unstimulated *Pfkfb3^*wt/wt*^* BMDMs. **(I)** *Pfkfb3* mRNA expression in *Pfkfb3^*fl/fl*^* relative to *Pfkfb3^*wt/wt*^* bone marrow-derived dendritic cells. The graphs represent mean ± SEM. * *p* < 0.05, ** *p* < 0.01. Data were analyzed using Mann-Whitney U test. ECAR; extracellular acidification rate.

To study if myeloid cells were indeed responsible for the observed effects of systemic PFKFB3 inhibition, we generated *Ldlr^–/–^ LysMCre^+/–^Pfkfb3^*fl/fl*^* (*Pfkfb3^*fl/fl*^*) mice, using *Ldlr^–/–^ LysMCre^+/–^Pfkfb3^*wt/wt*^* mice as controls (*Pfkfb3^*wt/wt*^*). Partial myeloid *Pfkfb3* knockdown was confirmed in *Pfkfb3^*fl/fl*^* versus *Pfkfb3^*wt/wt*^* BMDMs (50%, Figure 2A). As available antibodies are non-specific, confirmation of PFKFB3 knockdown on a protein level was prevented. Therefore, we further sought to obtain functional confirmation of *Pfkfb3* knockdown. The (near-)complete inhibition of glycolysis (≥ 80%) induces cell death (Schoors et al., 2014). Thus, partial glycolysis inhibition is desirable to affect cell function, without compromising cell viability. Seahorse analysis after glucose dosing revealed decreased basal extracellular acidification rate (ECAR) in *Pfkfb3^*fl/fl*^* BMDMs compared to controls (Figure 2B), indicating partially decreased glycolytic rates. During glycolysis, glucose is metabolized into pyruvate. Pyruvate can either be utilized in the tricarboxylic acid cycle to generate ATP, or metabolized into organic acids such as lactate (Lunt and Heiden, 2011). As expected based on glycolysis disruption, residual glucose levels were increased, whereas lactate levels were decreased in *Pfkfb3^*fl/fl*^* BMDM-conditioned medium (Figures 2C,D).

We previously mentioned that pro-inflammatory macrophages rely on glycolysis for their energy supply (Galván-Peña and O’Neill, 2014). As *PFKFB3* silencing using siRNA previously reduced glycolysis and pro-inflammatory activation of human macrophages (Tawakol et al., 2015), we studied the effect of *Pfkfb3* knockdown on BMDM cytokine gene expression. Indeed, already in unstimulated *Pfkfb3^*fl/fl*^* BMDMs, we observed increased expression of anti-inflammatory *Il10* (Figure 2E). Thereafter, we stimulated *Pfkfb3^*fl/fl*^* and *Pfkfb3^*wt/wt*^* BMDMs with LPS and IFN-γ to mimic the plaque pro-inflammatory phenotype of these cells and showed that partial *Pfkfb3* knockdown was maintained (60%, Figure 2F). Moreover, pro-inflammatory *Il6* and *Il12b* expression were decreased in *Pfkfb3^*fl/fl*^* versus *Pfkfb3^*wt/wt*^* BMDMs after pro-inflammatory stimulation (Figures 2G,H). These results indicate a decreased pro-inflammatory profile in *Pfkfb3^*fl/fl*^* macrophages, and thus confirm a role of *Pfkfb3* in pro-inflammatory macrophage polarization.

In our mouse model, Cre-recombinase expression is under control of the *Lyz2* promoter. Compared to macrophages, monocytes and neutrophils, *Lyz2* gene expression is low in DCs (Supplementary Figure 1A). Nevertheless, we assessed if DCs were targeted by our model, as *Pfkfb3* expression was abundant in this cell type (Figures 1B,C). We differentiated DCs from bone marrow cells and confirmed protein expression of the DC marker CD11c by flow cytometry (Supplementary Figure 1B). As expected based on lower *Lyz2* gene expression, DCs were not targeted in our model, as *Pfkfb3* expression was unchanged between *Pfkfb3^*fl/fl*^* and *Pfkfb3^*wt/wt*^* DCs (Figure 2I).

### No Effect of Partial Myeloid *Pfkfb3* Disruption on Atherosclerosis

**FIGURE 3:**
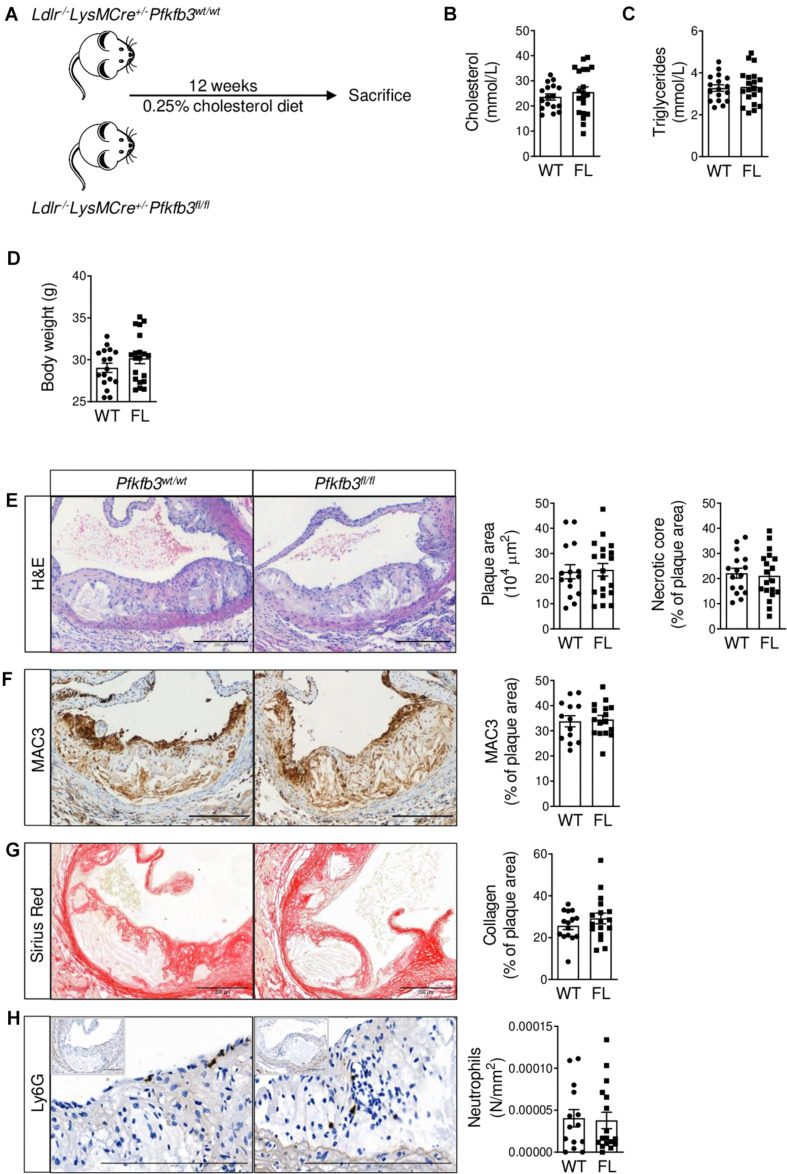
No effect of partial myeloid *Pfkfb3* disruption on advanced atherosclerotic lesions in aortic roots. **(A)** Setup of mouse experiment using *Ldlr^– /–^ LysMCre^+/–^Pfkfb3^*fl/fl*^* (*Pfkfb3^*fl/fl*^*, *n* = 20) and *Ldlr^– /–^ LysMCre^+/–^Pfkfb3^*wt/wt*^* (*Pfkfb3^*wt/wt*^*, *n* = 17) mice. **(B)** Cholesterol and **(C)** triglyceride levels in *Pfkfb3^*wt/wt*^* (WT) and *Pfkfb3^*fl/fl*^* (FL) plasma. **(D)** Body weight of *Pfkfb3^*wt/wt*^* and *Pfkfb3^*fl/fl*^* mice after 12 weeks of high cholesterol diet. **(E)** H&E, **(F)** MAC3, **(G)** Sirius Red and **(H)** Ly6G staining in *Pfkfb3^*wt/wt*^* and *Pfkfb3^*fl/fl*^* AR lesions and corresponding quantifications. The graphs represent mean ± SEM. Scale bars 200 μm. Data in B and H were analyzed using Mann-Whitney U test. Data in C–G were analyzed using Student’s *t*-test.

After confirming partial *Pfkfb3* knockdown, functional disruption of glycolysis and a decreased pro-inflammatory profile in macrophages *in vitro*, we studied the effects of myeloid *Pfkfb3* disruption on atherosclerosis. Therefore, *Pfkfb3^*fl/fl*^* and *Pfkfb3^*wt/wt*^* mice were fed a HCD for 12 weeks. We observed advanced atherosclerotic plaques in ARs with a necrotic core and fibrous cap (Figure 3A). Body weight and plasma cholesterol and triglyceride levels were similar between *Pfkfb3^*fl/fl*^* and *Pfkfb3^*wt/wt*^* mice after HCD (Figures 3B–D).

Unexpectedly, plaque and necrotic core size, as well as plaque macrophage and collagen content were unaffected in *Pfkfb3^*fl/fl*^* advanced AR lesions compared to controls (Figures 3E–G). Moreover, Ly6G+ neutrophil content was also unchanged between *Pfkfb3^*wt/wt*^* and *Pfkfb3^*fl/fl*^* AR lesions (Figure 3H). Similarly, no changes in plaque or necrotic core size were observed in early lesions without or with very little necrosis in BCA (Supplementary Figure 2A). Besides plaque size, total plaque burden, as measured by plaque index (Perrotta et al., 2020), was also unaffected in *Pfkfb3^*fl/fl*^* BCA (Supplementary Figure 2B).

### *Pfkfb* Isoenzyme Expression in Plaque Myeloid Cells

**FIGURE 4:**
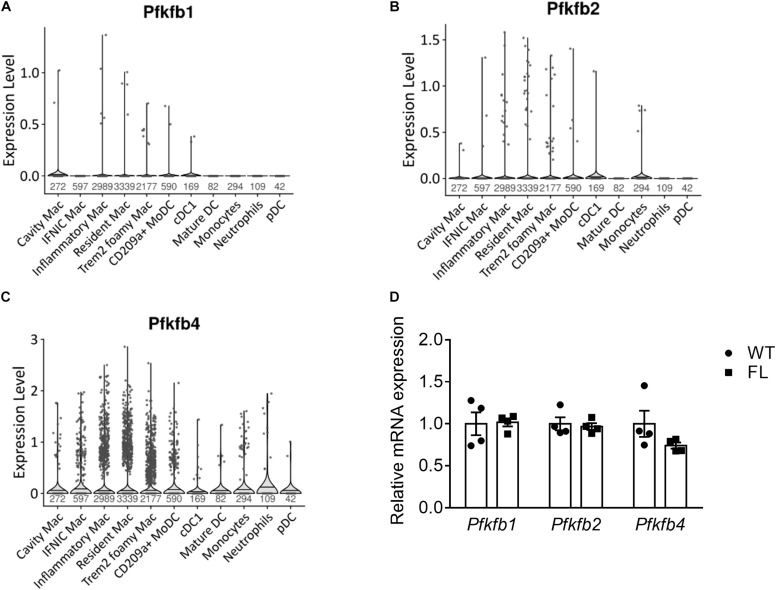
Expression of *Pfkfb* isoenzymes in plaque cells. Violin plot of **(A)** *Pfkfb1*, **(B)** *Pfkfb2*, and **(C)** *Pfkfb4* expression in murine atherosclerotic myeloid cells (Zernecke et al., 2020). **(D)** *Pfkfb1, Pfkfb2*, and *Pfkfb4* mRNA expression in *Pfkfb3^*fl/fl*^* (FL) relative to *Pfkfb3^*wt/wt*^* (WT) BMDMs. Data in **(D)** were analyzed using Two-way ANOVA. Sample sizes per cell type indicated under violin plots. CD, cluster of differentiation; cDC, conventional dendritic cell; IFNIC, interferon-inducible; Mac, macrophage; moDC, monocyte-derived dendritic cell; pDC, plasmacytoid DC; TREM2, triggering receptor expressed on myeloid cells 2.

To study potential genetic compensation by other *Pfkfb* isoenzymes keeping glycolytic rate above a certain threshold, we assessed expression of these isoenzymes in murine plaque myeloid cells. Expression of *Pfkfb1* and *Pfkfb2* was minimal in myeloid cells of the meta-analysis (Figures 4A,B) and *Ldlr^–/–^ LysMCre^+/–^* datasets (Supplementary Figures 3A,B). Similarly to *Pfkfb3*, *Pfkfb4* was expressed in macrophage and DC subsets, monocytes and neutrophils, albeit in a small proportion of cells (Figure 4C and Supplementary Figure 3C). To assess possible genetic compensation, we determined expression of *Pfkfb1, Pfkfb2*, and *Pfkfb4* in *Pfkfb3^*fl/fl*^* versus *Pfkfb3^*wt/wt*^* BMDMs, which was unaffected (Figure 4D). Thus, genetic compensation by other *Pfkfb* isoenzymes seems absent in BMDMs.

## Discussion

The current study assessed the effect of partial myeloid *Pfkfb3* disruption on atherosclerosis *in vivo*, after 12 weeks of HCD. Collectively, our findings suggest that although myeloid *Pfkfb3* disruption decreases the pro-inflammatory macrophage profile *in vitro*, it does not affect atherosclerosis development *in vivo*, neither in advanced, nor early lesions. No effects on circulating lipids, plaque size and composition, or total plaque burden were observed.

A few studies have looked into partial pharmacological inhibition of glycolysis in atherosclerosis by targeting PFKFB3, using 3PO(-derivatives). Similar to the current study, no effect on plaque size was reported (Beldman et al., 2019; Perrotta et al., 2020; Poels et al., 2020). Although plaque size was unchanged, total plaque burden over the aorta length was reduced in 3PO-treated *ApoE^–/–^* and *ApoE^–/–^ Fbn1^*C*1039G±^* mice (Perrotta et al., 2020). This decreased plaque occurrence was independent of changes in plaque composition, such as macrophage content, necrosis, fibrosis or angiogenesis.

In contrast, other studies did report effects of 3PO treatment on plaque composition. Plaque stability was increased, as indicated by decreased necrotic core area and a thicker fibrous cap, in *Ldlr^–/–^* mice treated with 3PO-derivative PFK158 (Poels et al., 2020). While Perrotta et al. (2020), hypothesized that decreased plaque burden after 3PO treatment was linked to decreased expression of EC adhesion molecules during early lesion development, no changes in EC adhesion molecules were observed in PFK158-treated *Ldlr^–/–^* mice (Poels et al., 2020). It was suggested that glycolysis inhibition in macrophages and monocytes could be responsible for the observed plaque stabilization.

On the contrary, here, we show that partially decreased PFKFB3-mediated glycolysis in monocytes, macrophages and granulocytes does not affect atherogenesis. Possibly, opposing effects of *Pfkfb3* knockdown within myeloid cells and subsets, result in an absence of net effect. However, we did not observe changes in neutrophil and macrophage numbers. Thus, positive effects reported after systemic 3PO treatment are likely conferred by other myeloid or stromal cell types, that are affected by inhibition of PFKFB3-mediated glycolysis and are important in atherogenesis, such as DCs, SMCs and fibroblasts. Indeed, we show that our model does not induce *Pfkfb3* knockdown in DCs. However, *Pfkfb3* expression is high in atherosclerotic DCs, and DCs play a fundamental role in atherogenesis by contributing to activation of adaptive immunity, foam cell formation and pro-inflammatory cytokine secretion (Zhao et al., 2021). Next to DCs and other myeloid cells, through analysis of scRNA-seq datasets, we showed that fibroblasts and SMCs, but also ECs and lymphocytes express *PFKFB3/Pfkfb3* in human and murine atherosclerosis. Importantly, increased αSMA + cells, i.e., SMCs and fibroblasts, were observed upon PFK158-treatment in *Ldlr^–/–^* and upon 3PO-treatment in *ApoE^–/–^* mice (Beldman et al., 2019; Poels et al., 2020). Additionally, EC activation and dysfunction are at the center of atherogenesis, while ECs also highly depend on glycolysis (De Bock et al., 2013; Eelen et al., 2015). Both specific *PFKFB3/Pfkfb3* knockdown in ECs and 3PO treatment reduced EC sprouting *in vivo* and *in vitro*, by affecting EC migration and proliferation (De Bock et al., 2013; Schoors et al., 2014). Moreover, 3PO decreased EC activation and increased endothelial barrier stability *in vitro*. However, while increasing plaque stability, 3PO treatment in *ApoE^–/–^* mice did not affect plaque endothelial barrier function (Beldman et al., 2019). Except for myeloid-specific *Pfkfb3* knockdown in the current study, effects of other cell-specific *Pfkfb3* knockdowns in atherosclerosis have not been studied yet. This could shine additional light on cell-specific effects of disrupted PFKFB3-mediated glycolysis on atherogenesis.

Another factor that might explain the lack of effect on atherosclerosis compared to studies utilizing 3PO treatment, is recent evidence that 3PO inhibits glycolysis through intracellular acidification, rather than specific PFKFB3 inhibition (Burmistrova et al., 2019; Emini Veseli et al., 2020). Thus, one should take possible unintended off-target effects of intracellular acidification into consideration when using 3PO(-derivatives). Small molecule AZ67 does bind to PFKFB3 specifically (Boyd et al., 2015; Emini Veseli et al., 2020) and might be an interesting pharmacological inhibitor for future *in vivo* atherosclerosis studies, while keeping in mind that effects are likely not mediated by monocytes, macrophages or neutrophils.

In addition to greater relevance of PFKFB3-mediated glycolysis in other cell types in atherosclerosis, or off-target effects of reported inhibitors, other factors may explain the observed lack of effect of myeloid *Pfkfb3* inhibition on atherosclerosis.

Firstly, *Pfkfb3* knockdown in *Pfkfb3^*fl/fl*^* BMDMs is only partial (∼50–60%). The LysMCre-loxP system often results in ≥ 70% deletion efficiency in myeloid cells (Clausen et al., 1999). Efficiency of the Cre-lox system in our model could be complicated by *Pfkfb3* gene locus (Murray and Wynn, 2011). Moreover, it should be noted that although we report expression of *Pfkfb3* in atherosclerotic myeloid cells, the percentage of monocytes, neutrophils and macrophages that express *Pfkfb3* is low (∼10–20%, Supplementary Figure 4A). Furthermore, as PFKFB3 is merely one of several stimulators of glycolytic flux (Mor et al., 2011), *Pfkfb3* inhibition reduces glycolysis only partially, in line with previous studies that targeted PFKFB3-mediated glycolysis (De Bock et al., 2013; Schoors et al., 2014; Yetkin-Arik et al., 2019). Nevertheless, glycolysis inhibition by 3PO treatment *in vivo* is also partial (Schoors et al., 2014; Poels et al., 2020), and a similar, partial approach was very successful to change EC function *in vivo* (De Bock et al., 2013; Beldman et al., 2019).

Indeed, we focus only on PFKFB3-mediated glycolysis in the current study. Atherosclerotic plaques are associated with increased glycolytic activity (Ali et al., 2018). As glycolysis is controlled at different levels, other glycolytic regulators than PFKFB3 might be involved in this association, such as hexokinase 2, glucose transporter 1 or enolase 2, which should be studied in the future (Berg et al., 2002; Ali et al., 2018).

Another factor that could explain the lack of effect, is the possible role of other PFKFB isoenzymes. Although we showed that *Pfkfb1*, *Pfkfb2*, and *Pfkfb4* expression was unaffected in *Pfkfb3^*fl/fl*^* BMDMs, PFKFB isoenzyme activity could still be increased, independent of expression (Macut et al., 2019).

Finally, differences in experimental setup, gender, HCD length and composition, and vascular sites assessed may cause differences in observed effects between the current and previous studies (Supplementary Table 2; VanderLaan et al., 2004; Getz and Reardon, 2006; Man et al., 2020). Moreover, glycolysis inhibition using chronic gene silencing by LysMCre from embryonic stage versus acute pharmacological protein inhibition or siRNA silencing in adult mice may result in different functional outcomes and may also explain a lack of effect in the current study (Knight and Shokat, 2007). As mentioned, the selectivity of pharmacological agents is often not entirely clear.

In conclusion, we showed that partial myeloid knockdown of PFKFB3 does not affect atherosclerosis development. Positive effects of systemic, partial glycolysis inhibition on lesion stabilization or total plaque burden that were previously reported, might be conferred by other *Pfkfb3-*expressing cells such as DCs, fibroblasts, SMCs and lymphocytes. Possibly, more severe reduction of myeloid glycolysis may be needed.

## Data Availability Statement

The datasets presented in this study can be found in online repositories. The names of the repository/repositories and accession number(s) can be found in the article/Supplementary Material.

## Ethics Statement

The animal study was reviewed and approved by the regulatory authority of the Maastricht University Medical Centre.

## Author Contributions

JS, JDB, LT, and RT devised and planned the experiments. JDB, KVK, and RT carried out the experiments. JDB, KVK, MG, and RT performed the data analysis. JP-P, JS-R, YG, and KL were responsible for single-cell sequencing data analysis. PC kindly provided *Pfkfb3^*lox/lox*^* mice for the experiments and provided critical input to the manuscript. RT and JS wrote the manuscript. All authors reviewed and approved the manuscript.

## Conflict of Interest

The authors declare that the research was conducted in the absence of any commercial or financial relationships that could be construed as a potential conflict of interest.

## Publisher’s Note

All claims expressed in this article are solely those of the authors and do not necessarily represent those of their affiliated organizations, or those of the publisher, the editors and the reviewers. Any product that may be evaluated in this article, or claim that may be made by its manufacturer, is not guaranteed or endorsed by the publisher.

## Footnotes

1
https://saezlab.github.io/dorothea/

## Abbreviations

3PO: 3-(3-pyridinyl)-1-(4-pyridinyl)-2-propen-1-one
ApoE: apolipoprotein E
BMDM: bone marrow-derived macrophages
CD: cluster of differentiation
cDC: conventional dendritic cell
DAB: diaminobenzidine
DC: dendritic cell
EC: endothelial cell
ECAR: extracellular acidification rate
H&E: haematoxylin and eosin
HCD: high cholesterol diet
HIF: hypoxia inducible factor
IFN: interferon
LCM: L929-conditioned medium
LDLr: low density lipoprotein receptor
Ly6G: lymphocyte antigen 6 complex locus G6D
LysM: lysozyme M
(ox)LDL: oxidized low density lipoprotein
moDC: monocyte-derived dendritic cell
NET: neutrophil extracellular trap
pDC: plasmacytoid dendritic cell
PFK-1: phosphofructokinase-1
PFKFB: 6-phosphofructo-2-kinase/fructose-2;6-biphosphatase
ROS: reactive oxygen species
scRNA-seq: single-cell RNA sequencing
SMC: smooth muscle cell
TREM2: triggering receptor expressed on myeloid cells 2.

## References

[B1] Ali L. Schnitzler J. G. Kroon J. (2018). Metabolism: the road to inflammation and atherosclerosis. *Curr. Opin. Lipidol.* 29 474–480. 10.1097/mol.0000000000000550 30234554

[B2] Bankhead P. Loughrey M. B. Fernández J. A. Dombrowski Y. McArt D. G. Dunne P. D. (2017). QuPath: open source software for digital pathology image analysis. *Sci. Rep.* 7:16878. 10.1038/s41598-017-17204-5 29203879 PMC5715110

[B3] Beldman T. J. Malinova T. S. Desclos E. Grootemaat A. E. Misiak A. L. S. van der Velden S. (2019). Nanoparticle-Aided Characterization of Arterial Endothelial Architecture during Atherosclerosis Progression and Metabolic Therapy. *ACS Nano* 13 13759–13774. 10.1021/acsnano.8b08875 31268670 PMC6933811

[B4] Berg J. M. Tymoczko J. L. Stryer L. (2002). *Biochemistry*, 5th Edn. New York: W H Freeman.

[B5] Boyd S. Brookfield J. L. Critchlow S. E. Cumming I. A. Curtis N. J. Debreczeni J. (2015). Structure-Based Design of Potent and Selective Inhibitors of the Metabolic Kinase PFKFB3. *J. Med. Chem.* 58 3611–3625. 10.1021/acs.jmedchem.5b00352 25849762

[B6] Burmistrova O. Olias-Arjona A. Lapresa R. Jimenez-Blasco D. Eremeeva T. Shishov D. (2019). Targeting PFKFB3 alleviates cerebral ischemia-reperfusion injury in mice. *Sci. Rep.* 9:11670. 10.1038/s41598-019-48196-z 31406177 PMC6691133

[B7] Clausen B. E. Burkhardt C. Reith W. Renkawitz R. Förster I. (1999). Conditional gene targeting in macrophages and granulocytes using LysMcre mice. *Transgenic Res.* 8 265–277. 10.1023/a:1008942828960 10621974

[B8] De Bock K. Georgiadou M. Schoors S. Kuchnio A. Wong B. W. Cantelmo A. R. (2013). Role of PFKFB3-Driven Glycolysis in Vessel Sprouting. *Cell* 154 651–663. 10.1016/j.cell.2013.06.037 23911327

[B9] Eelen G. de Zeeuw P. Simons M. Carmeliet P. (2015). Endothelial cell metabolism in normal and diseased vasculature. *Circ. Res.* 116 1231–1244. 10.1161/CIRCRESAHA.116.302855 25814684 PMC4380230

[B10] Eisenbarth S. C. (2019). Dendritic cell subsets in T cell programming: location dictates function. *Nat. Rev. Immunol.* 19 89–103. 10.1038/s41577-018-0088-1 30464294 PMC7755085

[B11] Emini Veseli B. Perrotta P. Van Wielendaele P. Lambeir A. M. Abdali A. Bellosta S. (2020). Small molecule 3PO inhibits glycolysis but does not bind to 6-phosphofructo-2-kinase/fructose-2,6-bisphosphatase-3 (PFKFB3). *FEBS Lett.* 594 3067–3075. 10.1002/1873-3468.13878 32620030

[B12] Faust N. Varas F. Kelly L. M. Heck S. Graf T. (2000). Insertion of enhanced green fluorescent protein into the lysozyme gene creates mice with green fluorescent granulocytes and macrophages. *Blood* 96 719–726. 10887140

[B13] Galván-Peña S. O’Neill L. A. J. (2014). Metabolic reprograming in macrophage polarization. *Front. Immunol.* 5:420. 10.3389/fimmu.2014.00420 25228902 PMC4151090

[B14] Garcia-Alonso L. Holland C. H. Ibrahim M. M. Turei D. Saez-Rodriguez J. (2019). Benchmark and integration of resources for the estimation of human transcription factor activities. *Genome Res.* 29 1363–1375. 10.1101/gr.240663.118 31340985 PMC6673718

[B15] Getz G. S. Reardon C. A. (2006). Diet and Murine Atherosclerosis. *Arterioscler. Thromb. Vasc. Biol.* 26 242–249. 10.1161/01.ATV.0000201071.49029.17 16373607

[B16] Holland C. H. Tanevski J. Perales-Patón J. Gleixner J. Kumar M. P. Mereu E. (2020). Robustness and applicability of transcription factor and pathway analysis tools on single-cell RNA-seq data. *Genome Biol.* 21:36. 10.1186/s13059-020-1949-z 32051003 PMC7017576

[B17] Knight Z. A. Shokat K. M. (2007). Chemical Genetics: where Genetics and Pharmacology Meet. *Cell* 128 425–430. 10.1016/j.cell.2007.01.021 17289560

[B18] Kumar S. Dikshit M. (2019). Metabolic Insight of Neutrophils in Health and Disease. *Front. Immunol.* 10:2099. 10.3389/fimmu.2019.02099 31616403 PMC6764236

[B19] Lunt S. Y. Heiden M. G. V. (2011). Aerobic Glycolysis: meeting the Metabolic Requirements of Cell Proliferation. *Annu. Rev. Cell Dev. Biol.* 27 441–464. 10.1146/annurev-cellbio-092910-154237 21985671

[B20] Macut H. Hu X. Tarantino D. Gilardoni E. Clerici F. Regazzoni L. (2019). Tuning PFKFB3 Bisphosphatase Activity Through Allosteric Interference. *Sci. Rep.* 9:20333. 10.1038/s41598-019-56708-0 31889092 PMC6937325

[B21] Man J. J. Beckman J. A. Jaffe I. Z. (2020). Sex as a Biological Variable in Atherosclerosis. *Circ. Res.* 126 1297–1319. 10.1161/CIRCRESAHA.120.315930 32324497 PMC7185045

[B22] Mantovani A. Garlanda C. Locati M. (2009). Macrophage Diversity and Polarization in Atherosclerosis. *Arterioscler. Thromb. Vasc. Biol.* 29 1419–1423. 10.1161/ATVBAHA.108.180497 19696407

[B23] Moore K. J. Tabas I. (2011). Macrophages in the pathogenesis of atherosclerosis. *Cell* 145 341–355. 10.1016/j.cell.2011.04.005 21529710 PMC3111065

[B24] Mor I. Cheung E. C. Vousden K. H. (2011). Control of Glycolysis through Regulation of PFK1: old Friends and Recent Additions. *Cold Spring Harb. Symp. Quant. Biol.* 76 211–216. 10.1101/sqb.2011.76.010868 22096029

[B25] Murray P. J. Wynn T. A. (2011). Obstacles and opportunities for understanding macrophage polarization. *J. Leukoc. Biol.* 89 557–563. 10.1189/jlb.0710409 21248152 PMC3058818

[B26] Perrotta P. Veken B. V. D. Veken P. V. D. Pintelon I. Roosens L. Adriaenssens E. (2020). Partial Inhibition of Glycolysis Reduces Atherogenesis Independent of Intraplaque Neovascularization in Mice. *Arterioscler. Thromb. Vasc. Biol.* 40 1168–1181. 32188275 10.1161/ATVBAHA.119.313692 PMC7176341

[B27] Poels K. Schnitzler J. G. Waissi F. Levels J. H. M. Stroes E. S. G. Daemen M. (2020). Inhibition of PFKFB3 Hampers the Progression of Atherosclerosis and Promotes Plaque Stability. *Front. Cell Dev. Biol.* 8:581641. 10.3389/fcell.2020.581641 33282864 PMC7688893

[B28] Schoors S. De Bock K. Cantelmo A. R. Georgiadou M. Ghesquière B. Cauwenberghs S. (2014). Partial and transient reduction of glycolysis by PFKFB3 blockade reduces pathological angiogenesis. *Cell Metab.* 19 37–48. 10.1016/j.cmet.2013.11.008 24332967

[B29] Silvestre-Roig C. Braster Q. Ortega-Gomez A. Soehnlein O. (2020). Neutrophils as regulators of cardiovascular inflammation. *Nat. Rev. Cardiol.* 17 327–340. 10.1038/s41569-019-0326-7 31996800

[B30] Stuart T. Butler A. Hoffman P. Hafemeister C. Papalexi E. Mauck W. M. III (2019). Comprehensive Integration of Single-Cell Data. *Cell* 177 1888–1902.e21. 10.1016/j.cell.2019.05.031 31178118 PMC6687398

[B31] Subramanian M. Tabas I. (2014). Dendritic cells in atherosclerosis. *Semin. Immunopathol.* 36 93–102. 10.1007/s00281-013-0400-x 24196454 PMC3946524

[B32] Tabas I. Williams K. J. Borén J. (2007). Subendothelial Lipoprotein Retention as the Initiating Process in Atherosclerosis. *Circulation* 116 1832–1844. 10.1161/CIRCULATIONAHA.106.676890 17938300

[B33] Tawakol A. Singh P. Mojena M. Pimentel-Santillana M. Emami H. MacNabb M. (2015). HIF-1α and PFKFB3 Mediate a Tight Relationship Between Proinflammatory Activation and Anerobic Metabolism in Atherosclerotic Macrophages. *Arterioscler. Thromb. Vasc. Biol.* 35 1463–1471. 10.1161/atvbaha.115.305551 25882065 PMC4441599

[B34] van Kuijk K. Demandt J. A. F. Perales-Patón J. Theelen T. L. Kuppe C. Marsch E. (2021). Deficiency of myeloid PHD proteins aggravates atherogenesis via macrophage apoptosis and paracrine fibrotic signalling: atherogenic effects of myeloid PHD knockdown. *Cardiovasc. Res.* [Epub Online ahead of print]. 10.1093/cvr/cvab152 33913468 PMC8953448

[B35] VanderLaan P. A. Reardon C. A. Getz G. S. (2004). Site Specificity of Atherosclerosis. *Arterioscler. Thromb. Vasc. Biol.* 24 12–22. 10.1161/01.ATV.0000105054.43931.f0 14604830

[B36] Wculek S. K. Khouili S. C. Priego E. Heras-Murillo I. Sancho D. (2019). Metabolic Control of Dendritic Cell Functions: digesting Information. *Front. Immunol.* 10:775. 10.3389/fimmu.2019.00775 31073300 PMC6496459

[B37] Willemsen L. de Winther M. P. (2020). Macrophage subsets in atherosclerosis as defined by single-cell technologies. *J. Pathol.* 250 705–714. 10.1002/path.5392 32003464 PMC7217201

[B38] Wirka R. C. Wagh D. Paik D. T. Pjanic M. Nguyen T. Miller C. L. (2019). Atheroprotective roles of smooth muscle cell phenotypic modulation and the TCF21 disease gene as revealed by single-cell analysis. *Nat. Med.* 25 1280–1289. 10.1038/s41591-019-0512-5 31359001 PMC7274198

[B39] Yetkin-Arik B. Vogels I. M. C. Nowak-Sliwinska P. Weiss A. Houtkooper R. H. Van Noorden C. J. F. (2019). The role of glycolysis and mitochondrial respiration in the formation and functioning of endothelial tip cells during angiogenesis. *Sci. Rep.* 9:12608. 10.1038/s41598-019-48676-2 31471554 PMC6717205

[B40] Zernecke A. (2015). Dendritic Cells in Atherosclerosis. *Arterioscler. Thromb. Vasc. Biol.* 35 763–770. 10.1161/ATVBAHA.114.303566 25675999

[B41] Zernecke A. Winkels H. Cochain C. Williams J. W. Wolf D. Soehnlein O. (2020). Meta-Analysis of Leukocyte Diversity in Atherosclerotic Mouse Aortas. *Circ. Res.* 127 402–426. 10.1161/circresaha.120.316903 32673538 PMC7371244

[B42] Zhao Y. Zhang J. Zhang W. Xu Y. (2021). A myriad of roles of dendritic cells in atherosclerosis. *Clin. Exp. Immunol.* [Epub Online ahead of print]. 10.1111/cei.13634 34109619 PMC8446417

