# Supplementary material for: Partial Inhibition of the 6-Phosphofructo-2-Kinase/Fructose-2,6-Bisphosphatase-3 (PFKFB3) Enzyme in Myeloid Cells Does Not Affect Atherosclerosis
Source: Front Cell Dev Biol. 2021 Aug 12;9:695684. doi: 10.3389/fcell.2021.695684 (PMC8387953; doi:10.3389/fcell.2021.695684)
Supplement: Supplementary file 1 [file Data_Sheet_1.docx]

Supplementary Material

Supplementary Methods

**Flow cytometry dendritic cells**

Flow cytometry was performed to confirm cluster of differentiation (CD)11c protein expression in bone marrow-derived dendritic cells. Fc receptors were blocked (CD16/CD32 antibody, 1:100, 14-0161, Invitrogen) and LIVE/DEAD Fixable Aqua Stain was used to assess viability (1:1000, L34957, Invitrogen). Thereafter, cells were stained with a CD11c antibody (PE-Cy7 conjugated, 1:1000, 25-0114, Invitrogen). Flow cytometry samples were measured with a BD FACSCanto II and analyzed with BD FACSDiva Software Version 6.1.2 (BD Biosciences).

**Supplementary Figures**


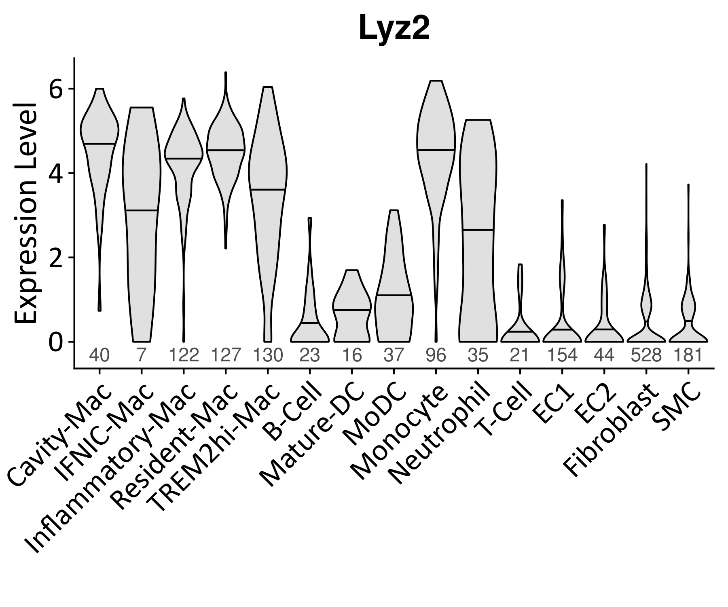

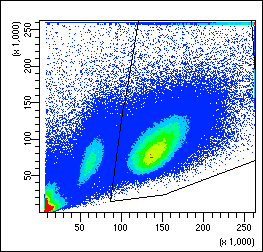

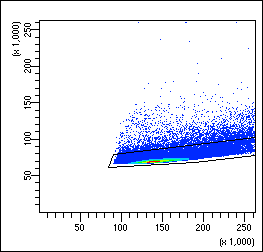

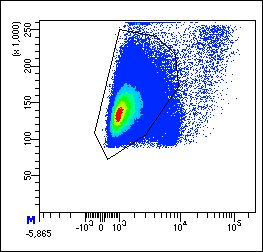

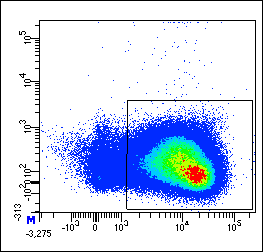

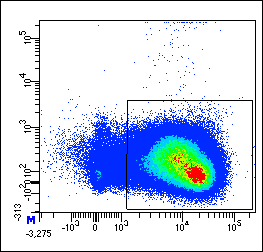


**A**

**B**

**FSC-A**

**FSC-W**

**SSC-A**

**Singlets**

**Live**

**Live/dead stain**

**FSC-A**

**FSC-A**

***Pfkfb3^fl/fl^***

***Pfkfb3^wt/wt^***

**91.0%**

**91.1%**

**CD11c**

**CD11c**

**Supplementary Figure 1: Expression of *Lyz2* in *Ldlr^-/-^LysMCre^+/-^* plaque cells and confirmation of protein expression of CD11c in bone marrow-derived dendritic cells**

**(A)** Violin plot of *Lyz2* expression in single cell populations from murine *Ldlr^-/-^LysMCre^+/-^* aortic arch lesions (Van Kuijk et al., 2021). Sample sizes per cell type indicated under violin plots. **(B)** Flow cytometry gating strategy and percentages of CD11c positive cells of living *Pfkfb3^wt/wt^* and *Pfkfb3^fl/fl^* bone marrow-derived dendritic cells. EC; endothelial cell, IFNIC; interferon-inducible, Lyz2; lysozyme 2, Mac; macrophage, moDC; monocyte-derived dendritic cell, SMC; smooth muscle cell, TREM2; triggering receptor expressed on myeloid cells 2


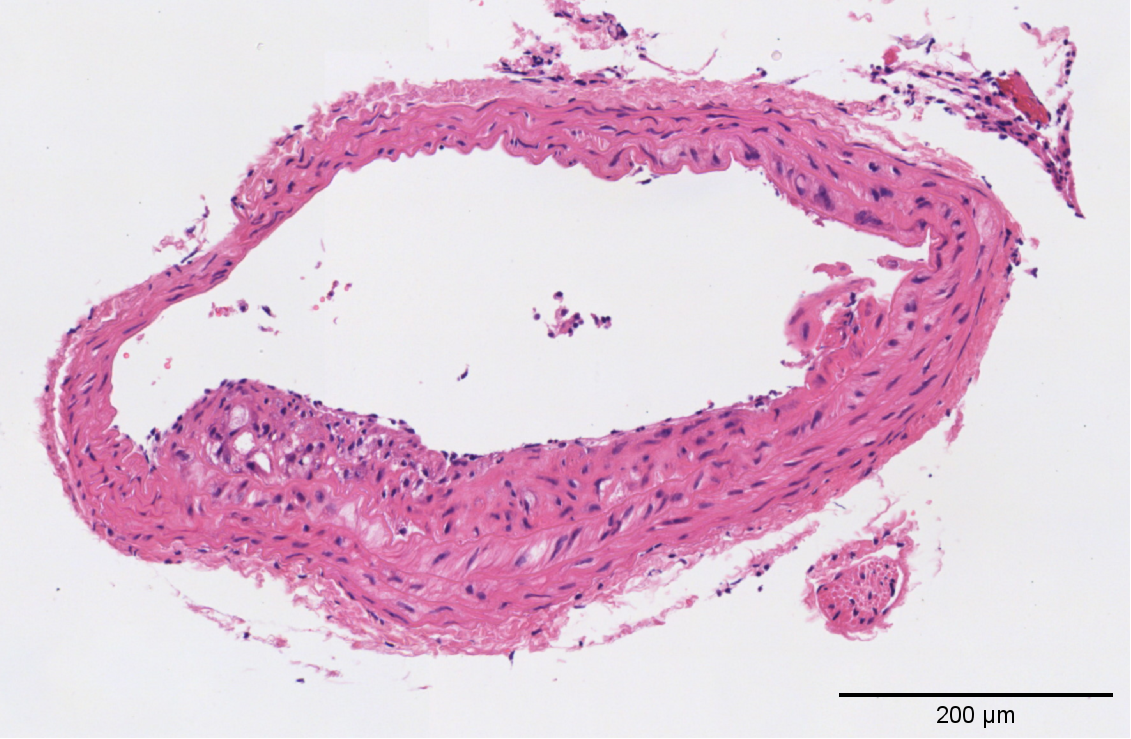

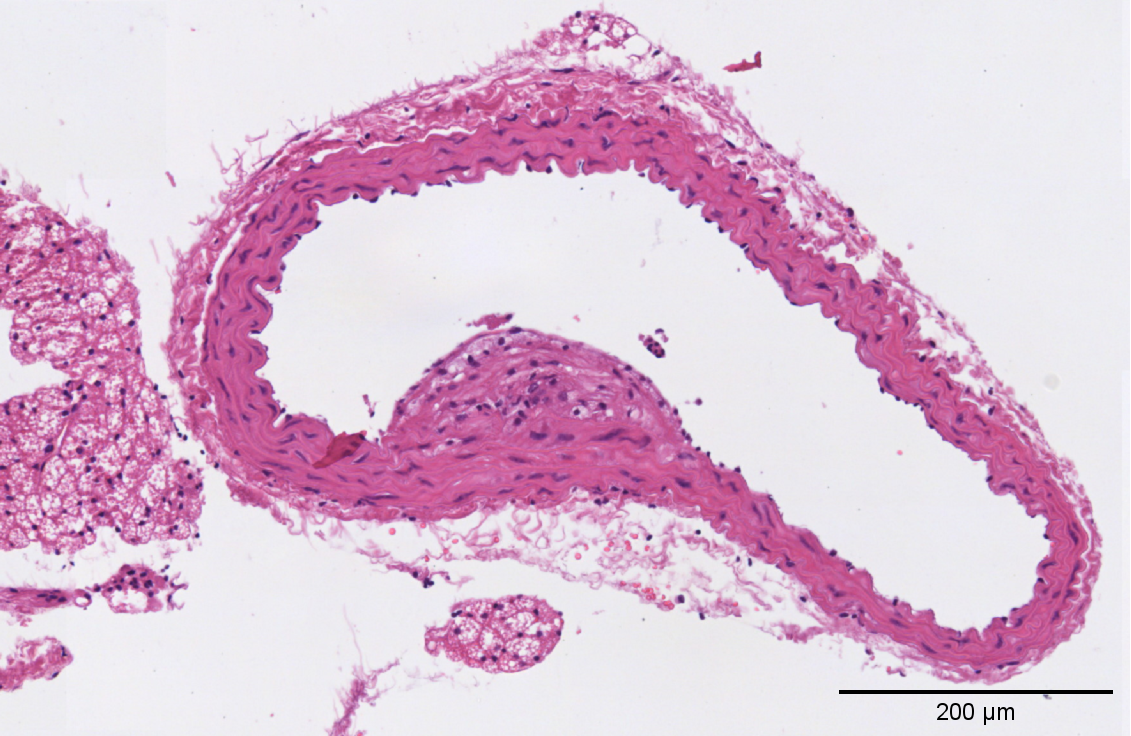


*Pfkfb3^fl/fl^*

*Pfkfb3^wt/wt^*

**B**

H&E

**A**

**Supplementary Figure 2: No effect of partial myeloid *Pfkfb3* disruption on early atherosclerotic lesions in brachiocephalic arteries**

**(A)** H&E staining in *Pfkfb3^wt/wt^* (WT) and *Pfkfb3^fl/fl^* (FL) brachiocephalic artery lesions and corresponding quantifications. **(B)** Total plaque burden in *Pfkfb3^wt/wt^* and *Pfkfb3^fl/fl^* brachiocephalic arteries. The graphs represent mean ± SEM. Scale bars 200 µm. Data were analyzed using Mann-Whitney U test. BCA; brachiocephalic artery


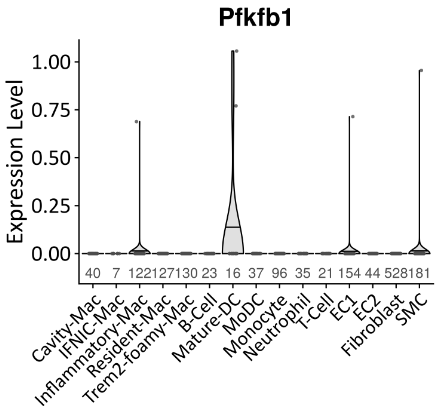

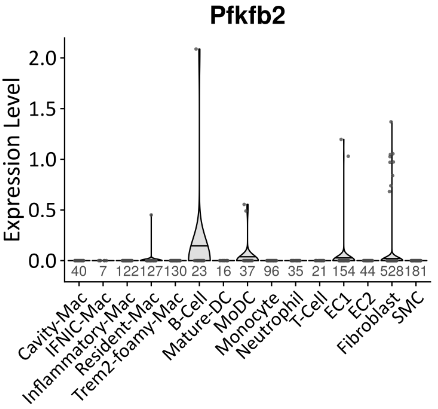

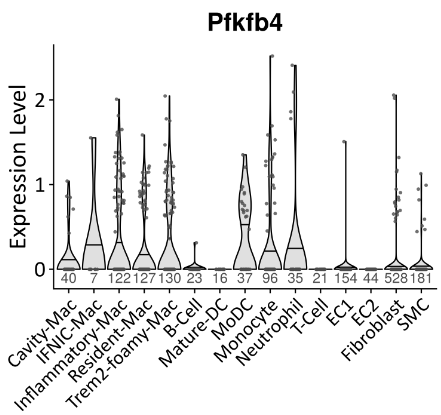


**A**

**B**

**C**

**Supplementary Figure 3: Expression of *Pfkfb* isoforms in *Ldlr^-/-^LysMCre^+/-^* plaque cells**

Violin plot of **(A)** *Pfkfb1*, **(B)** *Pfkfb2* and **(C)** *Pfkfb4* expression in single cell populations from murine *Ldlr^-/-^LysMCre^+/-^* aortic arch lesions (Van Kuijk et al., 2021). Sample sizes per cell type indicated under violin plots. EC; endothelial cell, IFNIC; interferon-inducible, Mac; macrophage, moDC; monocyte-derived dendritic cell, SMC; smooth muscle cell, TREM2; triggering receptor expressed on myeloid cells 2

**Ldlr^-/-^LysMCre^+/-^**

**A**


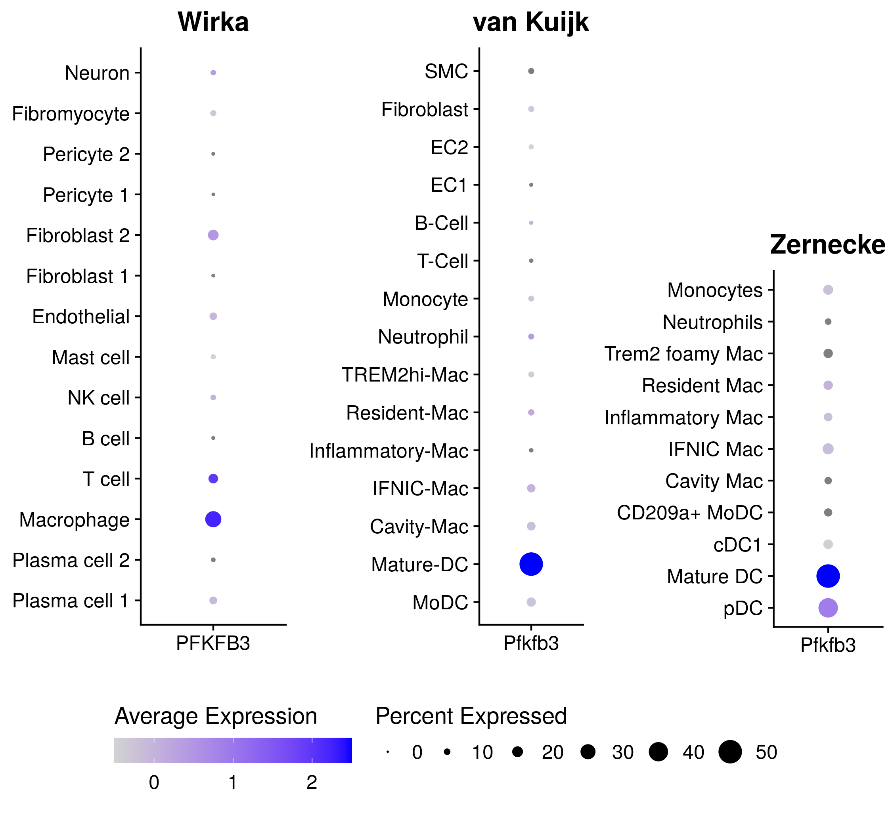


**Human coronary atherosclerosis**

**Meta-analysis**

**Supplementary Figure 4: Dot plots of *PFKFB3*/*Pfkfb3* expression in human and murine atherosclerosis**

**(A)** Dot plots of *PFKFB3/Pfkfb3* expression in single cell populations of human atherosclerotic coronary arteries (Wirka et al., 2019), murine *Ldlr^-/-^LysMCre^+/-^* aortic arch lesions (Van Kuijk et al., 2021) and murine atherosclerotic aorta (meta-analysis, Zernecke et al., 2020). CD; cluster of differentiation, cDC; conventional dendritic cell, EC; endothelial cell, IFNIC; interferon-inducible, Lyz2; lysozyme 2, Mac; macrophage, moDC; monocyte-derived DC, NK cell; natural killer cell, pDC; plasmacytoid DC, SMC; smooth muscle cell, TREM2; triggering receptor expressed on myeloid cells 2

**Supplementary Table 1: Primer sets used for qPCR**

| **Gene** | **Forward primer (5’-3’)** | **Reverse primer (5’-3’)** |
| --- | --- | --- |
| *18s* *rRNA* | GTAACCCGTTGAACCCCATT | CCATCCAATCGGTAGTAGCG |
| *Pfkfb1* | AGCCTTTGGATGAGGAATTG | GTGTGCCCACATCGAAGAT |
| *Pfkfb2* | AATGAGATTGATGCTGGCGTG | ATTCCTCTGGGTACCGTTGC |
| *Pfkfb3* | CTATCCCACGGGAGAGTCC | TGGCGCTCTAATTCCATGA |
| *Pfkfb4* | AACTGACCCAGAATCCCCTG | GTTAGTCATGCAGACACCACG |

**Supplementary Table 2: Characteristics and outcomes of glycolysis inhibition studies in murine atherosclerosis.**

| **Study** | **Intervention** | **Model** | **Gender** | **Composition/length diet** | **Sites assessed** | **Effect on plaque size/index** | **Effect on plaque composition** |
| --- | --- | --- | --- | --- | --- | --- | --- |
| Current | *LysMCre^+/-^Pfkfb3^-/-^* | *Ldlr^-/-^* | M | 0.25% cholesterol, 12 weeks | AR, BCA | Size =  Index = | Necrotic core =  Macrophages =  Collagen =  MCs N/A |
| Poels et al., 2020 | PFK158  *2 µg/g*  *5 wks*  *3x/wk* | *Ldlr^-/-^* | M | 0.15% cholesterol, 13 weeks | AR, AA | Size =  Index N/A | Necrotic core ↓  Macrophages =  Collagen N/A  MCs ↑ |
| Perrotta et al., 2020 | 3PO  *50 µg/g* *preventive:* *10 wks*  *2x/wk* *curative:*  *4 wks*  *4x/wk* | *ApoE^-/-^* | F | 0.15% cholesterol, 14-20 weeks | CA, AA | Size =  Index ↓ | Necrotic core =  Macrophages =  Collagen =  MCs = |
| Beldman et al., 2019 | 3PO  *25 µg/g*  *6 wks*  *3x/wk* | *ApoE^-/-^* | F | 0.2% cholesterol, 6 weeks | AR | Size =  Index N/A | Necrotic core =  Macrophages ↓  Collagen ↑  MCs ↑ |

M; male, F; female, AR; aortic root, BCA; brachiocephalic artery, AA; aortic arch, CA; carotid artery, N/A; not assessed, MCs; mesenchymal cells, =; unchanged, ↑; increased, ↓; decreased.
